# Effect of tragacanth gum–chitin nanofiber film containing free or nano‐encapsulated cumin essential oil on the quality of chilled turkey burgers packed with oxygen absorber

**DOI:** 10.1002/fsn3.4202

**Published:** 2024-05-14

**Authors:** Nasim Shahabi, Aziz A. Fallah, Masoud Sami, Saeid Habibian Dehkordi

**Affiliations:** ^1^ Department of Food Hygiene and Quality Control, Faculty of Veterinary Medicine Shahrekord University Shahrekord Iran; ^2^ Department of Food Science and Technology, School of Nutrition and Food Science, Food Security Research Center Isfahan University of Medical Sciences Isfahan Iran; ^3^ Department of Basic Sciences, Faculty of Veterinary Medicine Shahrekord University Shahrekord Iran

**Keywords:** active packaging, biodegradable film, muscle food, nanocomposite film, nanoemulsion

## Abstract

This research was undertaken to assess the effect of tragacanth gum–chitin nanofiber (TG–CNF) film containing free (CEO) or encapsulated cumin essential oil (CNE) combined with oxygen absorber (OA) packaging on the shelf‐life of ready‐to‐cook (RTC) turkey breast burgers during chilled storage. The experimental groups were OA and TG–CNF as single treatments, TG–CNF + CEO, TG–CNF + CNE, and TG–CNF + OA as binary treatments, TG–CNF + CEO + OA and TG–CNF + CNE + OA as ternary treatments, and control. The samples were stored at 3°C for 20 days and analyzed for microbial, physicochemical, and sensory attributes. Binary treatments, when compared to single treatments, and ternary treatments, when compared to binary treatments, exhibited enhanced effectiveness in managing microbial growth, hindering physicochemical alterations, and decelerating sensory alterations. At day 20, TG–CNF + CNE + OA group was identified as the most effective group in inhibiting the growth of total mesophilic bacteria (TMB), total psychrophilic bacteria (TSB), and coliforms (final counts were 4.8, 4.16, and ≤1 log CFU/g, respectively), and TG–CNF + CNE + OA and TG–CNF + CEO + OA groups were known as the most effective groups in inhibiting lactic acid bacteria (LAB) (final counts were 4.71 and 5.15 log CFU/g, respectively). Furthermore, the TG–CNF + CNE + OA treatment proved to be the most effective group in reducing the total volatile nitrogen (TVN) (final level was 19.2 mg N/100 g) and thiobarbituric acid reactive substances (TBARS) (final level was 0.119 mg malondialdehyde (MDA)/kg). TG–CNF + CNE + OA and TG–CNF + CEO + OA were the most efficient groups to delay the increasing rate of cooking loss (final values were 23.3% and 24.6%) and pH (final values were 7.01 and 6.99). The sample's shelf‐life was 4 days in control and TG–CNF, 8 days in OA and TG–CNF + OA, 12 days in TG–CNF + CEO, 16 days in TG‐CNF + CNE and TG–CNF + CEO + OA, and at least 20 days in TG–CNF + CNE + OA. As a result, the incorporation of TG–CNF + CNE alongside OA packaging emerges as a highly effective active packaging method for preserving RTC turkey breast burgers during chilled storage.

## Graphical abstract

This research was undertaken to assess the effect of tragacanth gum–chitin nanofiber (TG–CNF) film containing free (CEO) or encapsulated cumin essential oil (CNE) combined with oxygen absorber (OA) packaging on the shelf life of ready‐to‐cook (RTC) turkey breast burgers during chilled storage. The treated samples were stored at 3°C for 20 days and analyzed for microbial, physicochemical, and sensory attributes. The incorporation of TG–CNF + CNE alongside OA packaging emerges as a highly effective active packaging method for preserving RTC turkey breast burgers during chilled storage.

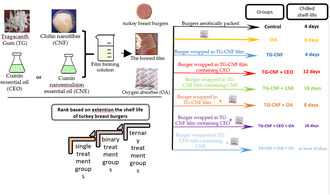

## 1 INTRODUCTION

Nowadays, the employment of biopolymer‐based active packaging has emerged as a premier approach to not only enhance food safety but also mitigate the environmental hazards associated with nonbiodegradable packaging materials. The polysaccharides, proteins, or lipid biopolymers are extensively employed to formulate the edible films because of their inherent properties, such as nontoxicity, degradability, and edibility (Alparslan et al., 2014; Li et al., 2023; Maroufi et al., 2023; Shahabi et al., 2023; Yavari Maroufi et al., 2022).

Tragacanth gum (TG), one of the diverse ranges of natural biopolymers, is a highly branched polysaccharide characterized by its anionic structure and the presence of protein fragments in small proportions. Two main components have been identified in the solution of this Asian‐originated gum, basorin (insoluble or water‐swellable fraction) and tragacanthic acid (tragacanthin or soluble fraction) (Abdolmaleki et al., 2016; Boamah et al., 2023; Carpentier et al., 2021). The application of TG biopolymer as a packaging material has some drawbacks such as low physical properties and permeability; hence, the combined use of TG with the other biodegradable materials can improve the characteristics of the blended film (Zare et al., 2019).

Chitin, the second abundant biomaterial with linear structure, exists in crab and prawn shells. Poor performance and crystallinity of chitin can be improved by arranging its structure to nanofiber. Despite the limited water solubility observed in commercial chitin, homogeneous chitin nanofibers (CNFs) readily dissolve in water. Therefore, the incorporation of CNF can effectively enhance the mechanical, thermal, and inhibitory characteristics of edible films through proper interfacial interaction between active structure of the film matrix and CNF. However, these properties are not the specific properties of the CNF (Chang et al., 2010; Ifuku, 2014; Sahraee et al., 2017).

Nanoemulsions, either water‐in‐oil or oil‐in‐water, are emulsions with nanoscale structures that can be created through low‐ or high‐energy techniques. In recent years, there has been a substantial increase in the utilization of nanoemulsions across the food industry, cosmetics, and pharmaceutical sectors due to their advantages, such as better stability, resistance to gravitational separation, and ability to provide a translucent appearance (Bagheri et al., 2016; Das et al., 2020; Javadian et al., 2017; Mahmud et al., 2022; Salvia‐Trujillo et al., 2017; Sayyari et al., 2021).

Oxygen absorbers (OAs) are designed to retard oxygen‐dependent food spoilage via iron (Fe^2+^) oxidation and reduce the oxygen levels inside the package to less than 0.01% (Cichello, 2015; Mexis & Kontominas, 2010). As one of the most popular active packaging methods, it is extensively utilized to retard undesirable oxidative processes by eliminating oxygen (Cichello, 2015).

There are some researches investigating the use of natural polymers in combination with cumin essential oil as active packaging to preserve muscle foods (Abdou et al., 2018; Rafiee et al., 2019; Takma & Korel, 2019; Xin et al., 2020), and no studies have reviewed the difference of free and nano‐encapsulated form of cumin essential oil on a food model. Hence, the objective of this research is to evaluate the impact of a nanocomposite film composed of gum–chitin nanofiber (TG–CNF) along with CEO or CNE, combined with OA packaging, on the quality and shelf life of chilled ready‐to‐cook (RTC) turkey breast burgers. Moreover, the comparison of free and nano‐encapsulated form of cumin essential oil on the quality of turkey burgers was investigated.

## 2 MATERIALS AND METHODS

### 2.1 Materials

Oxygen absorber packets (model 215WFG4540) with 300 cc oxygen absorption capacity were obtained from Total Packaging‐System Guardian Co. (Gimpo‐si, Korea). CNF (nanofiber diameter: 20–50 nm) was achieved from Nano‐Novin Polymer Co. (Gorgan, Iran). High‐quality TG was purchased from a local store (Isfahan, Iran). Pure cumin essential oil was procured from Dorrin Golab Co. (Kashan, Iran). Low‐density polyethylene/ethylene vinyl alcohol–polyamide copolymer/polyethylene terephthalate (LDPE/EVOH/PET) high oxygen barrier pouches (110 μm thickness and oxygen transmission rate of ~2.1 mL/m^2^/24 at 25°C) were obtained from Plastic Machine Alvan Co. (Karaj, Iran). The chemicals used in this research were of analytical grade, indicating their superior quality and purity. Microbial culture media, such as plate count agar (PCA), violet red bile glucose agar (VRBGA), and de Man–Rogosa–Sharpe (MRS), were supplied by MirMedia Company (Khorramshahr, Iran).

### 2.2 Cumin essential oil analysis

The chemical composition analysis of the purified CEO was conducted using gas chromatography–mass spectrometry (GC–MS), following the methodology outlined by Sarmast et al. (2019). The compounds were identified based on GC retention indices and computer matching with the Wiley library, as well as by comparison of the mass spectra with those reported in the literature (Adams, 2001).

### 2.3 Preparation and characterization of CNE

The CNE was prepared by mixing CEO and Tween 80 (1:1 (*v*/*v*)) and then subjected to ultrasonic homogenization for 15 min at a power output of 450 W and frequency of 20 kHz (model UP400‐A, TOP Sonics Co., Tehran, Iran) (Dini et al., 2020). Herein, the final concentration of essential oil was fixed at 6%. Crashed ice was placed around the emulsion beaker to decrease the generated heat during the ultrasonication process. The average particle size and polydispersity index (PDI) of CNE droplets were measured by using SZ‐100 dynamic light scattering (DLS) (Horiba, Japan). Finally, the CNE was carefully preserved in a sealed glass bottle at a temperature of 4°C. Throughout a period of 8 weeks, the emulsion underwent regular monitoring on a weekly basis to evaluate any potential separation of its components.

### 2.4 Preparation of tragacanth film

Initially, the TG fragments were immersed in distilled water for a duration of 24 h. Following this, glycerol was introduced, constituting 50% of the dry weight of the TG. The mixture was then subjected to ultrasonic homogenization using a power output of 400 for a total duration of 10 min (Raoufi et al., 2019). Subsequently, the solution was cooled to room temperature, and then the mixture received the addition of CNF at a concentration of 5% w/w based on the dry weight of TG. The mixture was carefully stirred to ensure thorough blending and dispersion of the CNF. Based on our initial investigation, it was found that the TG film containing 5% CNF (w/w of dry weight of biopolymer) exhibited the most favorable mechanical and physical properties. Moreover, the properties of the films containing CNE and CEO are presented in Appendix S1. The essential oil emulsion (free essential oil) or nanoemulsion (encapsulated essential oil) was added to the solution. The TG was ultimately present in the solution at a concentration of 1% w/v, while the essential oil, whether free or encapsulated, was also present at a concentration of 1%. Subsequently, the solution was transferred to steel trays for drying purposes, employing a temperature of 40°C.

### 2.5 Sample preparation

Turkey breasts (6 h after slaughter) were obtained from a local poultry company (Samaneh Co., Isfahan, Iran) and were promptly transferred to the laboratory while maintaining a cold chain throughout the transportation process. Upon arrival, samples were deboned and washed with chilled water (1–2°C). Sunflower oil (4%), chopped onion (6%), toasted flour (8%), mixed spices (1%), and salt (1%) were added to minced meat according to the national standard of Iran (“Institute of Standards and Industrial Research of Iran, Chicken burger – preparation procedure,” 2021). Next, the pre‐prepared burgers (5 cm diameter, ~1.5 cm thickness) were randomly assigned to eight different groups, as outlined in Table 1. The experimental groups were OA and TG–CNF as single treatments, TG–CNF + CEO, TG–CNF + CNE, and TG–CNF + OA as binary treatments, TG–CNF + CEO + OA and TG–CNF + CNE + OA as ternary treatments, and control. The samples were stored at 3°C for 20 days and analyzed for microbial, physicochemical, and sensory attributes. All of the samples were carefully stored at a temperature of 3 ± 0.2°C. Subsequently, microbiological, physicochemical, and sensory analyses were conducted at specific time intervals of 0, 4, 8, 12, 16, and 20 days. These analyses were performed in four replications for each group, totally 192 specimens were analyzed.

**TABLE 1:** Experimental groups and their descriptions.

Group No.	Treatment	Description
1	Control	Burger samples aerobically packed
2	OA	Burger samples were packed with OA packet
3	TG–CNF	Burger samples wrapped in TG–CNF film and then aerobically packed
4	TG–CNF + CEO	Burger samples wrapped in TG–CNF film containing CEO and then aerobically packed
5	TG–CNF + CNE	Burger samples wrapped in TG–CNF film containing CNE and then aerobically packed
6	TG–CNF + OA	Burger samples wrapped in TG–CNF film and then packed with OA packet
7	TG–CNF + CEO + OA	Burger samples wrapped in TG–CNF films containing CEO and then packed with OA packet
8	TG–CNF + CNE + OA	Burger samples wrapped in TG–CNF films containing CNE and then packed with OA packet

Abbreviations: CEO, cumin essential oil; CNE, cumin nanoemulsion; OA, oxygen absorber; TG–CNF, tragacanth gum–chitin nanofiber.

### 2.6 Microbiological analyses

An amount of 10 g of RTC turkey breast burger and 90 mL of sterile normal saline (0.85% NaCl) were added to a sterile bag aseptically. The content was homogenized by BagMixer 400 (Interscience, Mourjou, France), and serial decimal dilutions were prepared. Enumeration of total mesophilic bacteria (TMB) and total psychrophilic bacteria (TSB) on plate count agar was done after incubation at 35°C for 48 h and 7°C for 10 days, respectively. Also, the enumeration of lactic acid bacteria (LAB) on MRS agar and coliforms on violet red blue agar (VRBGA) was performed after incubation at 35°C for 48 h (Fallah et al., 2008).

### 2.7 Physicochemical analyses

The total volatile nitrogen (TVN), thiobarbituric acid reactive substances (TBARS), and pH were determined according to the previously published methods (Dini et al., 2020; Fallah et al., 2010; Melero et al., 2012; Shen et al., 2015).

#### 2.7.1 Cooking loss

The cooking loss was assessed, according to the method previously described (Barbanti & Pasquini, 2005; Trindade et al., 2013). In brief, the pre‐prepared burger samples were weighted individually and placed in trays. Subsequently, the trays were placed in an electric oven set at a temperature of 180°C. Following the cooking process, the samples were allowed to cool down to 25°C and then reweighed. The cooking loss was determined using the following equation:

$$\text{Cooking loss}\;\left(\%\right)=\frac{A-B}{A}\times 100$$

Where *A* and *B* denote uncooked and cooked sample weights, respectively.

### 2.8 Sensory analyses

Sensory evaluation of cooked samples was carried out by the panelists who had experience in turkey burger assessment. Assessors evaluated the coded samples in individual places, randomly. In this vein, the taste, color, odor, and texture of cooked samples were investigated using a 7‐point hedonic scale. The scale was defined as follows: 1 = very poor, 2 = poor, 3 = slight, 4 = fair, 5 = moderate, 6 = good, and 7 = excellent. The scores below four were unacceptable. The mean scores (color, taste, odor, and texture) were determined as overall acceptability (Uran & Yilmaz, 2017).

### 2.9 Statistical analysis

The SPSS software (version 22) was used to analyze the obtained experimental data. The two‐way analysis of variance (ANOVA) followed by the Duncan post hoc test was employed to investigate the effect of storage time and treatments on the measured parameters. The *p* ≤ .050 was considered significant.

## 3 RESULTS AND DISCUSSION

### 3.1 Specifications of CEO and CNE

The analysis of the CEO demonstrated the presence of 15 compounds representing 99.59% of the total identified components. Terpinene (11.16%), phellandrene (11.16%), menthatriene (11.06%), terpineol (11.06%), cuminaldehyde (11.06%), and carvyl acetate (11.06%) were recognized as the main components (Table 2). In this vein, Dini et al. (2020) and Homayonpour et al. (2021) found that γ‐terpinene, cumin aldehyde, and cuminic alcohol were the highest concentration compounds of CEO, which is consistent with our result. In another study, β‐pinene and ρ‐cymene were showed as the major compounds that were not matching with our result (Ravi et al., 2013).

**TABLE 2:** Chemical composition of cumin (*Cuminum cyminum*) essential oil.

No.	Compound	Retention index	Composition (%)
1	Heptanal	801	6.98
2	Terpinene	1011	11.16
3	Limonene	1028	3.31
4	Phellandrene	1053	11.16
5	γ‐Terpinene	1056	4.05
6	p‐Cymene	1069	4.04
7	Linalool oxide	1074	2.11
8	Menthatriene	1100	11.06
9	Terpineol	1133	11.06
10	Thujanol	1152	5.43
11	Piperitone	1254	1.93
12	Cuminaldehyde	1323	11.06
13	Carvyl acetate	1361	11.06
14	α‐Terpinen‐7‐al	1364	1.7
15	Thymol	1545	3.48
	Total		99.59

The average particle size of cumin nanoemulsion was 26.1 nm with a polydispersity index (PDI) of 0.436. Over a period of 8 weeks at a temperature of 4°C, CNE exhibited consistent stability without any indications of creaming or phase separation. In the studies conducted by Dini et al. (2020) and Venkadesaperumal et al. (2016), it was found that the average particle diameter of cumin nanoemulsion was 89.61 and 52.89 nm.

### 3.2 Microbial analyses

**FIGURE 1:**
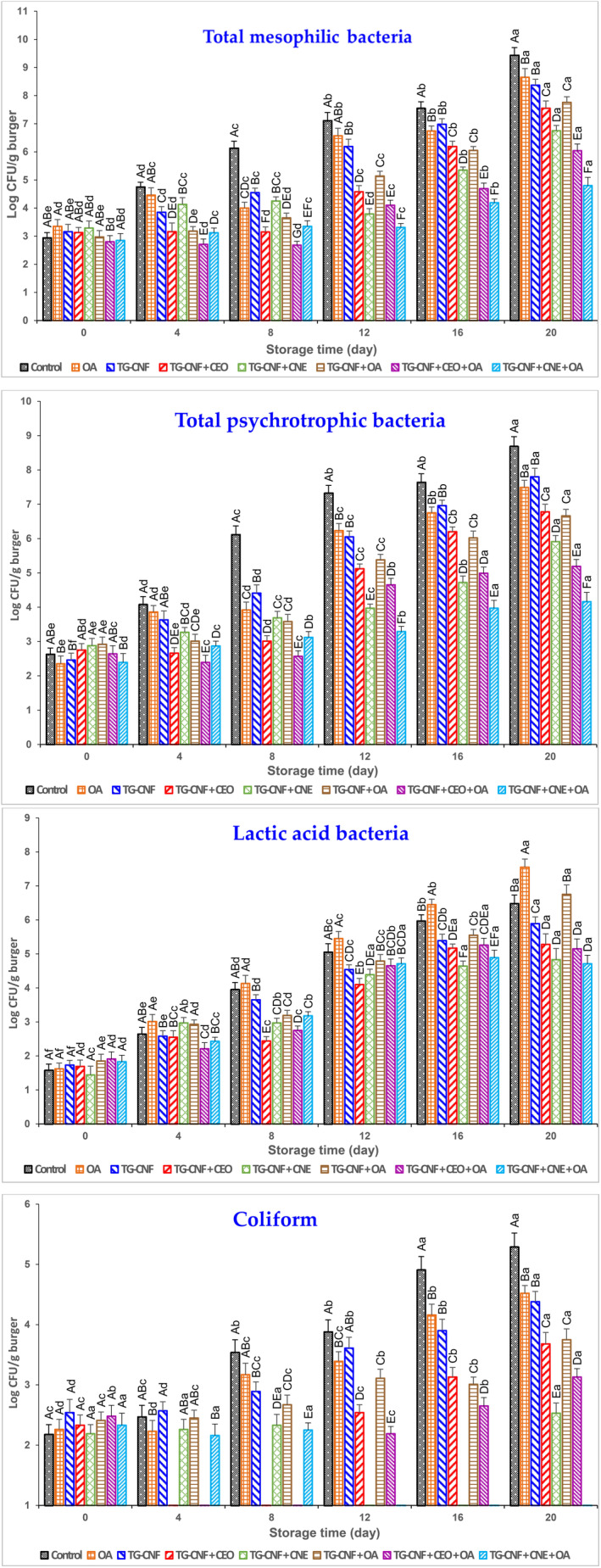
Microbial counts of RTC turkey breast burger treated with tragacanth gum–chitin nanofiber (TG–CNF) film containing cumin essential oil nanoemulsion (CNE) or cumin essential oil (CEO) and packed with oxygen absorber (OA) during refrigeration. The different capital letters (A–F) show significant differences among treatments at each day of the storage period, while lowercase letters (a–f) show significant differences among the storage days in the same treatment.

Figure 1 shows the counts of microbial flora in control and treated groups of turkey breast burgers during chilled storage. At day 0, no significant difference was observed in counts of TMB, TSB, and coliforms among the groups (*p* > .05); and the range of mentioned bacteria was 2.80–3.35, 2.35–2.92, and 2.10–2.50 log CFU/g, respectively. During the storage period, there was a significant increase in TMB, TSB, and coliforms in all groups (*p* ≤ .05). However, the growth rate of bacteria was observed to be slower in the treatment groups compared to the control group. The coliforms were not detected from days 4 to 8 in TG–CNF + CEO and TG–CNF + CEO + OA, 12 to 16 in TG–CNF + CNE, and 12 to the end of storage in TG–CNF + CNE + OA group. At day 20 (end of storage), TMB, TSB, and coliforms were significantly higher in the control group than in the other treated groups (*p* ≤ .05); and there was no significant difference between OA and TG–CNF, and also between TG–CNF + CEO and TG–CNF + OA groups (*p* > .05). The binary treatments (TG–CNF + CEO, TG–CNF + CNE, and TG–CNF + OA) were more effective than single treatments (OA and TG–CNF) to control the growth of TMB, TSB, and coliforms; and the TG–CNF + CNE had the lowest count compared to the other binary treatments.

Furthermore, among the various treatments, the TG–CNF + CNE + OA combination proved to be the most effective in inhibiting the growth of TMB, TSB, and coliforms. During the initial period from day 0 to 8, the treatments that included free essential oil (TG–CNF + CEO and TG–CNF + CEO + OA) demonstrated greater efficacy in reducing TMB, TSB, and coliforms counts compared to the treatments containing encapsulated essential oil (TG–CNF + CNE and TG–CNF + CNE + OA). However, from day 12 to 20, the treatments containing CNE exhibited higher effectiveness in reducing mentioned bacterial counts (Figure 1). Slower release of active compounds from encapsulated essential oil resulted in higher microbial flora count in the groups that contained essential oil nanoemulsion, as compared to the groups that contained free essential oil. Nonetheless, the sustained release of active compounds from the encapsulated essential oil led to a greater antimicrobial effect in the treatments that contained nanoemulsion compared to those that contained free essential oil, particularly after the initial days of storage (Pabast et al., 2018; Zhang et al., 2019; Zhao et al., 2023). In agreement with our result, Zhang et al. found that nano‐encapsulated *Paulownia tomentosa* essential oil showed enhanced effectiveness in reducing microbial counts in RTC pork compared to free essential oil by the conclusion of the storage period. When taking into account the maximum acceptable microbial limit for fresh poultry burgers (“Institute of Standards and Industrial Research of Iran, Raw frozen chicken‐burger – Specifications and test methods”, 2016), it was observed that the TMB levels exceeded the threshold of 6 log CFU/g at different time points. However, the TMB level of the TG–CNF + CNE + OA group remained below the standard limit throughout the storage period. Behbahani et al. (2020) indicated that the immersion of beef in *Lallemantia iberica* seed mucilage, enriched with cumin essential oil, led to a decrease in the growth rate of mesophilic and psychrophilic bacteria during refrigerated storage. In another study, olive oil nanoemulsion combined with ajowan essential oil was effective in reducing the counts of TMB in chilled ground chicken (Jafarinia et al., 2022).

The initial count of LAB was between 1.4 and 1.9 log CFU/g in experimental groups of RTC turkey breast burgers. Notably, there were no significant differences observed among the groups in terms of LAB count. It has been determined that LAB can grow both under aerobic and microaerophilic conditions (Kamkar et al., 2021). Our findings indicated that while the count of LAB was significantly higher in the OA group on day 20, there was no significant difference in LAB count between the treatments with or without OA.

Among the binary treated groups (TG–CNF + CEO, TG–CNF + CNE, and TG–CNF + OA), the TG–CNF + OA group exhibited the highest LAB count. This can be attributed to the inhibitory effect of the active compounds (CEO and CNE) on the growth of LAB. Moreover, there were no significant differences between the ternary treatments (TG–CNF + CEO + OA and TG–CNF + CNE + OA) at day 20. Giatrakou et al. (2010) demonstrated that combined chitosan–thyme treatments under modified atmosphere packaging conditions (30% carbon dioxide (CO_2_) and 70% nitrogen (N_2_)) decreased LAB counts in RTC poultry products during chilled storage.

Generally, the antimicrobial properties of the TG–CNF film are related to chitin. The antimicrobial action of chitin can be described by two mechanisms: (a) interaction between the positively charged amino groups (–NH3+) of chitin and the negatively charged groups (R‐COO‐, R‐OPO (O2)^2−^) present on the external surface of bacterial cells, which leads to the disruption of the bacterial cell membrane, ultimately causing cell death; (b) bacterial flocculation in the presence of chitin, which restricts the transfer of nutrients and oxygen to the bacterial cells, ultimately hindering bacterial growth and development (Shankar et al., 2015). The small particle size of CNF can play an essential role in enhancing antimicrobial properties. This phenomenon is related to a larger surface area and appropriate distribution of nanofiber of chitin in the film matrix (Sahraee et al., 2017; Salaberria et al., 2015). It has been revealed that the monoterpenic compounds in CEO, such as γ‐terpinene, cumin aldehyde, ρ‐cymene, limonene, and ß‐pinene, exert damaging effects on the cytoplasmic membrane of microorganisms. This damage causes the leakage of intracellular materials, eventually leading to the cell death (Hasani‐Javanmardi et al., 2021). By converting the essential oils to nanoscale particles, their effectiveness in transferring bioactive compounds through bacterial lipid membrane is significantly improved. This enhanced capability enables a more intensified antimicrobial effect (Salvia‐Trujillo et al., 2015).

### 3.3 Physicochemical analyses

**FIGURE 2:**
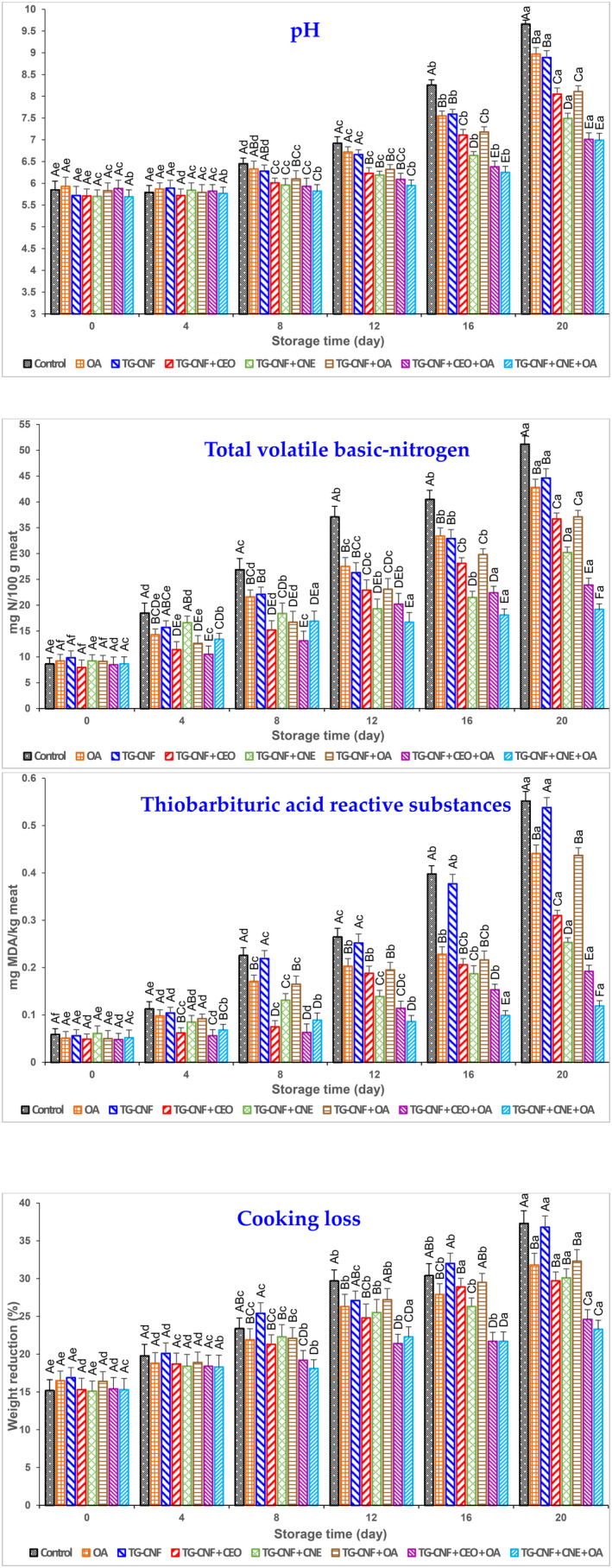
Physicochemical parameters' chart of RTC turkey breast burgers treated with tragacanth gum–chitin nanofiber (TG–CNF) film containing cumin essential oil nanoemulsion (CNE) or cumin essential oil (CEO) and packed with oxygen absorber (OA) during refrigeration. The different capital letters (A–F) show significant differences among treatments at each day of the storage period, while lowercase letters (a–f) show significant differences among the storage days in the same treatment.

The results of physicochemical analyses are presented in Figure 2. The TVN can detect a wide spectrum of volatile amines produced by bacterial proteolytic enzymes during the breakdown of proteins and nonprotein nitrogenous substances (Pirastehfard et al., 2021). According to Figure 2, the initial TVN values in the various experimental groups ranged from 7.95 to 9.84 mg N/100 g, indicating a high quality of RTC turkey breast burgers (*p* > .05). The TVN index exhibited an increase in all groups throughout the storage period. Notably, the control group demonstrated a higher rate of increase in the TVN index compared to the other groups. At day 20, ternary treatments (TG–CNF + CNE + OA and TG–CNF + CEO + OA) compared to binary treatments (TG–CNF + CEO, TG–CNF + OA, and TG–CNF + CNE) and binary treatments compared to single treatments (OA and TG–CNF) were more efficient to control the increasing rate of TVN. Therefore, the TG–CNF + CNE treatment was identified as the most effective among the binary groups in reducing TVN levels. Furthermore, the TG–CNF + CNE + OA treatment exhibited greater efficacy compared to the TG–CNF + CEO + OA treatment. As it is well known, the acceptable limit of TVN is 25 mg N/100 g. According to our findings, the TVN level exceeded the standard limit at day 8 in the control group, day 12 in the OA and TG–CNF groups, day 16 in the TG–CNF + CEO and TG‐CNF + OA groups, and day 20 in the TG–CNF + CNE group. Furthermore, the TG–CNF + CNE + OA treatment demonstrated greater effectiveness in controlling the TVN level compared to the TG–CNF + CEO + OA treatment at the end of the storage period. During the initial period from day 0 to 8, the treatments containing CEO (TG–CNF + CEO and TG–CNF + CEO + OA) exhibited lower TVN levels compared to the treatments containing encapsulated essential oil (TG–CNF + CNE and TG–CNF + CNE + OA). However, from day 8 to 20, the TVN levels of the treatments containing CNE were lower compared to levels of the treatments containing free CEO. The findings of our study revealed that the sustained and prolonged release of active compounds in the treatments containing encapsulated essential oil effectively suppressed microbial growth. Consequently, the production of TVN was significantly delayed throughout the storage period. Similar findings have been reported by Fathimoghadam et al. (2023) regarding refrigerated shrimp that were wrapped in gelatin biopolymer loaded with Persian lime essential oil.

The number of secondary products from lipid oxidation that produce undesirable off‐odor and flavor can be measured by the TBARS index (Hasani‐Javanmardi et al., 2021). Initially, on day 0, there were no notable distinctions in the TBARS level observed among the various groups. However, as the storage time progressed, the TBARS level exhibited a consistent upward trend across all groups. Notably, the control and TG–CNF groups demonstrated a more rapid increase in TBARS levels compared to the other treatments. At the end of storage, it was observed that the ternary treatments, specifically TG–CNF + CNE + OA and TG–CNF + CEO + OA, proved to be more effective in controlling lipid oxidation compared to the binary treatments (TG–CNF + CEO, TG–CNF + OA, and TG–CNF + CNE). Also, among the binary and ternary treatments, the TG–CNF + CNE and TG–CNF + CNE + OA groups demonstrated the highest efficacy in controlling lipid oxidation. TG–CNF + CNE + OA emerged as the superior group among all the treatment groups in terms of effectively regulating the TBARS level. Between days 0 and 8, the treatments incorporating free essential oil (TG–CNF + CEO and TG–CNF + CEO + OA) displayed greater efficacy in reducing lipid oxidation compared to the treatments containing encapsulated essential oil (TG–CNF + CNE and TG–CNF + CNE + OA). However, from day 8 to 20, the treatments with encapsulated essential oil surpassed those with free essential oil in terms of controlling lipid oxidation. The prolonged release of active compounds in the treatments containing encapsulated essential oil appears to have a beneficial effect during storage by slowing down lipid oxidation and thereby reducing the TBARS level (Bagheri et al., 2016). In this regard, a study conducted by Zhang et al. (2020) revealed that the application of nano‐encapsulated tarragon essential oil proved to be more effective in retarding lipid oxidation in pork slices when compared to the use of free essential oil. The antioxidant activity of essential oils is likely attributable to the presence of various monoterpenic hydrocarbons, such as γ‐terpinene, cumin aldehyde, limonene, and ρ‐cymene. These specific compounds possess the ability to effectively scavenge and neutralize free radicals, thus providing a plausible explanation for the essential oil's antioxidant properties (Dini et al., 2020; Hasani‐Javanmardi et al., 2021). By employing OA sachets to eliminate any remaining oxygen in burger packaging, the oxidative processes can be slowed down (Hasani‐Javanmardi et al., 2021). In this vein, Mohan et al. (2019) found that the vacuum or oxygen absorber packaging retarded the oxidative processes in Indian oil sardine during chilled storage.

There were no notable variations in the pH value across treatments on days 0 and 4 (*p* > .05). During the storage period, the pH value showed an upward trend in all groups. However, the rate of increase in the control group was significantly higher compared to the other groups (*p* ≤ .05). At day 20, the control group indicated the highest pH value (*p* ≤ .05). Following the control group, the single treatments (OA and TG–CNF) displayed notably higher pH values compared to the binary treatments (TG–CNF + CEO, TG–CNF + OA, and TG–CNF + CNE). Also, binary treatments showed higher pH values than ternary treatments (TG–CNF + CNE + OA and TG–CNF + CEO + OA). Among the binary groups, the TG–CNF + CNE treatment exhibited the lowest pH value. When considering the ternary treatments (TG‐CNF + CNE + OA and TG‐CNF + CEO + OA), there was no significant difference observed in the pH value at the end of the storage period (*p* > .05). In the study conducted by Homayonpour et al. (2021), it was observed that nano‐chitosan coating with nano‐encapsulated cumin essential oil resulted in a notable reduction in the rate of pH increase in sardine fillet.

On days 0 and 4, there were no significant differences among all groups regarding the cooking loss values. Throughout the storage period, all groups experienced an increase in cooking loss values; however, the control and TG–CNF groups displayed a significantly higher rate of increase in cooking loss compared to the other groups (*p* ≤ .05). At the end of the storage period, there were no significant differences between the control and TG–CNF groups, both of which had the highest cooking loss values. The most efficient groups were TG–CNF + CEO + OA and TG–CNF + CNE + OA; there was no significant difference between them in cooking loss values. It may be attributed to the decrease in the thermal protein denaturation of turkey burger tissue in mentioned groups during the cooking process, which leads to the improvement in the water‐holding capacity of meat. The possible reason behind the improved water‐holding capacity of the meat in the mentioned groups during the cooking process is a decrease in thermal protein denaturation within the turkey burger tissue. This decrease contributes to preserving the structural integrity of the proteins, allowing them to better retain moisture (Darwish et al., 2012). In this regard, the investigation carried out by Noori et al. (2018) demonstrated that the nanoemulsion‐based edible coating containing ginger essential oil was effective in reducing the cooking loss of chicken breast fillets.

### 3.4 Sensory analyses

**FIGURE 3:**
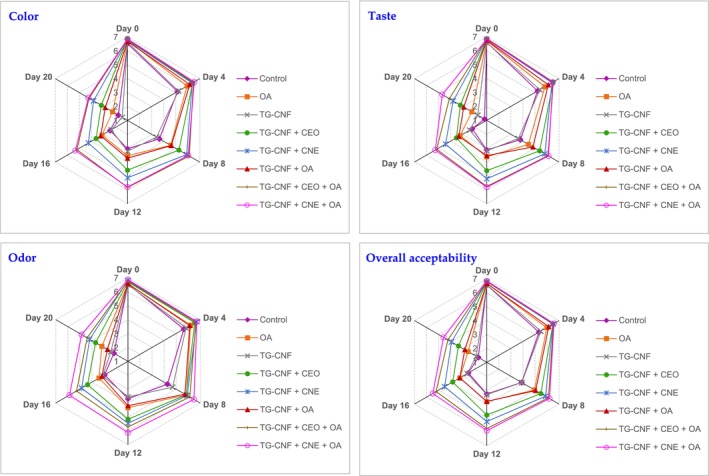
Sensory analyses of RTC turkey breast burger treated with tragacanth gum–chitin nanofiber (TG–CNF) film containing cumin essential oil nanoemulsion (CNE) or cumin essential oil (CEO) and packed with oxygen absorber (OA) during refrigeration.

Figure 3 demonstrates the results of sensory analyses including the appearance (color), odor, taste, and overall acceptability of burger samples. In the initial evaluation, both the control and treated groups received acceptable scores (score ≥ 6.51) for the assessed parameters. Throughout the storage period, both the TG–CNF + CEO + OA and TG–CNF + CNE + OA groups achieved satisfactory scores for all sensory characteristics. The samples received acceptable scores for color, taste, and overall acceptability for different durations: 4 days in both the control and TG–CNF groups, 8 days in the OA and TG–CNF + OA groups, 12 days in the TG–CNF + CEO group, and 16 days in the TG–CNF + CNE group. In terms of odor evaluation, acceptable scores were obtained for 8 days in both the control and TG–CNF groups, 12 days in the OA and TG–CNF + OA groups, 16 days in the TG–CNF + CEO group, and at least 20 days in the TG–CNF + CNE group. Considering multiple parameters, such as overall acceptability, total volatile basic nitrogen (TVB‐N), and TMB, the shelf‐life of the turkey breast samples varied across different treatments. The control and TG–CNF groups had a shelf life of 4 days, while the OA and TG–CNF + OA groups extended it to 8 days. The TG–CNF + CEO group demonstrated a shelf‐life of 12 days, while the TG–CNF + CNE and TG–CNF + CEO + OA groups reached 16 days. Notably, the TG–CNF + CNE + OA group exhibited a minimum shelf‐life of 20 days. Hasani‐Javanmardi et al. (2021) found that the application of CEO and safflower oil nanoemulsion combined with OA packaging improves the shelf‐life of lamb loins at 4°C .

## 4 CONCLUSIONS

The results of the present study indicated that utilizing an active film incorporating CNE or CEO in conjunction with oxygen absorber packaging effectively delayed bacterial growth and retarded physicochemical changes in RTC turkey breast burgers during refrigerated storage. The results revealed that the ternary treatment groups (TG–CNF + CEO + OA and TG–CNF + CNE + OA) exhibited greater effectiveness in extending the shelf‐life of turkey breast burgers compared to the binary treatment groups (TG–CNF + CEO, TG–CNF + CNE, and TG–CNF + OA). Similarly, the binary treatment groups were more effective in prolonging shelf‐life compared to the single treatment groups (OA and TG–CNF). Furthermore, it was observed that the films incorporating cumin essential oil nanoemulsion exhibited superior performance in controlling microbial growth and delaying the increase in TVN, TBARS, and cooking loss values in turkey burgers compared to the films containing free essential oil. As a result, the application of active TG–CNF film containing CNE or CEO followed by OA packaging emerges as one of the most effective active packaging approaches to enhance the shelf‐life of chilled RTC turkey breast burgers.

## AUTHOR CONTRIBUTIONS

**Nasim Shahabi:** Conceptualization (equal); data curation (equal); investigation (equal); methodology (equal); writing – original draft (equal). **Aziz A. Fallah:** Conceptualization (equal); data curation (equal); funding acquisition (equal); methodology (equal); software (equal); supervision (equal); validation (equal); writing – review and editing (equal). **Masoud Sami:** Conceptualization (equal); formal analysis (equal); writing – review and editing (equal). **Saeid Habibian Dehkordi:** Conceptualization (equal); resources (equal); writing – review and editing (equal).

## CONFLICT OF INTEREST STATEMENT

The authors have no conflicts of interest to declare.

## Supporting information


Appendix S1:

## Data Availability

Data available on request from the authors.
