# Supplementary material for: Effect of tragacanth gum–chitin nanofiber film containing free or nano‐encapsulated cumin essential oil on the quality of chilled turkey burgers packed with oxygen absorber
Source: Food Sci Nutr. 2024 May 14;12(8):5605–18. doi: 10.1002/fsn3.4202 (PMC11317702; doi:10.1002/fsn3.4202)
Supplement: Supplementary file 1 — Appendix S1: [file FSN3-12-5605-s001.docx]

**Supplementary Material**

**Appendix A**

**Determination of mechanical and physical properties of films**

***Methods***

The prepared films including TG, TG-CNF (1%, 3%, 5%, and 7% w/w of biopolymer dry weight), TG-CNF-CEO, and TG-CNF-CNE were conditioned at ~ 50% relative humidity for 48 h prior to the mechanical tests. The tensile strength (TS) and elongation at break (EB) of the films were determined using a universal testing machine (Santam, Karaj, Iran) according to the ASTM standard method D882 (Materials, 2002). The initial grip separation and cross-head speed were set at 70 mm and 50 mm/min, respectively. Three replicates were measured for each type of film.

The water vapor permeability (WVP) of the films was determined according to the method of (Casariego et al., 2009). The films were mechanically sealed on to the cells containing 2.5 g of calcium sulfate to create a 0% RH storage condition. The cells were initially weighed and placed in a desiccator containing saturated solution of potassium sulfate (97% RH at 25◦C), then the amount of water vapor transferred through the film and absorbed by the desiccant was determined from the weight gain of the cell. Weight gain of the permeation cell was periodically recorded every 2 h for 10 h. To calculate the water vapor transmission rate (WVTR), The slope of the weight vs. time plot was divided by the effective film area. The WVP was determined by the following formula:

WVP = (WVTR × X)/ΔP

Where, X is the film thickness and ΔP is the driving force.

***Results***

**Table A1.** Mechanical and physical properties of the films.

| Film | TS (MPa) | EB (%) | WVP(× 10^5^ g mm/m^2^ h Pa) |
| --- | --- | --- | --- |
| TG | 13.2 ± 0.11^a^ | 4.34 ± 0.12^a^ | 4.02 ± 0.08^a^ |
| TG-CNF 1% | 13.7 ± 0.13^b^ | 4.02 ± 0.09^b^ | 3.80 ± 0.10^b^ |
| TG-CNF 3% | 14.0 ± 0.07^c^ | 3.88 ± 0.16^c^ | 3.19 ± 0.08^c^ |
| TG-CNF 5% | 14.4 ± 0.10^d^ | 3.02 ± 0.09^d^ | 2.85 ± 0.05^d^ |
| TG-CNF 7% | 12.6 ± 0.19^e^ | 4.45 ± 0.06^e^ | 4.12 ± 0.07^a^ |
| TG-CNF-CEO | 13.9 ± 0.06^b^ | 3.88 ± 0.11^b^ | 2.04 ± 0.06^e^ |
| TG-CNF-CNE | 14.9 ± 0.11^f^ | 5.17 ± 0.07^f^ | 1.53 ± 0.09^f^ |

*Abbreviations:* TG, tragacanth gum; CNF, chitin nanofiber; CEO, cumin essential oil nanoemulsion; CNE, cumin essential oil nanoemulsion; TS, tensile strength, EB, elongation at break; WVP, water vapor permeability.

^a–f^ Different letters in the same column indicate significant difference (*P* < 0.050).

The TS of the TG-CNF films was significantly increased by increasing the CNF concentration up to 5%. It might be due to the proper interaction between the anionic film matrix and cationic nanoparticles (Rezaie, Rezaei, & Albooftileh, 2021; Sahraee, Milani, Ghanbarzadeh, & Hamishehkar, 2017). The TS of the TG-CNF film with 7% CNF was significantly decreased that might be due to the agglomeration and to some extent disintegration of the film structure. As a result, the CNF concentration of 5% (w/w of biopolymer dry weight) was selected for the preparation of TG-CNF-CEO and TG-CNF-CNE films. The incorporation of CNE to the film (TG-CNF-CNE) significantly increased the TS of the film. The film network may be reinforced through the extensive hydrogen bindings between polar groups present along polymer backbone and polar head groups of surfactant molecules present at the interface of dispersed nanodroplets (Sahraee et al., 2017).

The EB of the TG-CNF films was significantly decreased by increasing the CNF concentration up to 5% and increased again by more enhancing of CNF concentration (7%). Incorporation of the CNE to the TG-CNF film increased its EB that might be due to the plasticizing effect of the nanoemulsion. It has been reported that incorporating of essential oils may weaken the interactions between polymer chains hence results in more extensible and flexible films (Bilbao-Sáinz, Avena-Bustillos, Wood, Williams, & McHugh, 2010; Gahruie, Ziaee, Eskandari, & Hosseini, 2017).

The WVP of TG-CNF films was significantly decreased by increasing the CNF concentration up to 5%. It might be due to the creating the tortuous pass for crossing water vapor through the film, increasing the crystallinity of the biopolymer, or decreasing free hydrophilic groups in biopolymer matrix (Gahruie et al., 2017; Shankar, Reddy, Rhim, & Kim, 2015). The incorporation of CNE to the TG-CNF film decreased its WVP. The structure of Tween 80 in the nanoemulsion makes the polar outer membrane of the nanoparticles better responsive to the polymer chain, which improves the interdisciplinary coherence, reduces the paths and spaces and decreases the WVP. Moreover, the presence of uniform distributed and smaller nanoemulsion droplets in the film structure causes water molecules to pass through a curvilinear path that reduces the migration rate of water vapor molecules (Acevedo-Fani, Salvia-Trujillo, Rojas-Graü, & Martín-Belloso, 2015; Ghadetaj, Almasi, & Mehryar, 2018).

***References***

Acevedo-Fani, A., Salvia-Trujillo, L., Rojas-Graü, M. A., & Martín-Belloso, O. (2015). Edible films from essential-oil-loaded nanoemulsions: Physicochemical characterization and antimicrobial properties. *Food Hydrocolloids, 47*, 168-177.

Bilbao-Sáinz, C., Avena-Bustillos, R. J., Wood, D. F., Williams, T. G., & McHugh, T. H. (2010). Nanoemulsions prepared by a low-energy emulsification method applied to edible films. *Journal of Agricultural and Food Chemistry, 58*(22), 11932-11938.

Casariego, A., Souza, B., Cerqueira, M., Teixeira, J., Cruz, L., Díaz, R., & Vicente, A. (2009). Chitosan/clay films' properties as affected by biopolymer and clay micro/nanoparticles' concentrations. *Food Hydrocolloids, 23*(7), 1895-1902.

Gahruie, H. H., Ziaee, E., Eskandari, M. H., & Hosseini, S. M. H. (2017). Characterization of basil seed gum-based edible films incorporated with Zataria multiflora essential oil nanoemulsion. *Carbohydrate Polymers, 166*, 93-103.

Ghadetaj, A., Almasi, H., & Mehryar, L. (2018). Development and characterization of whey protein isolate active films containing nanoemulsions of Grammosciadium ptrocarpum Bioss. essential oil. *Food Packaging and Shelf Life, 16*, 31-40.

Materials, A. S. f. T. (2002). *Standard test method for tensile properties of thin plastic sheeting*: Astm International.

Rezaie, A., Rezaei, M., & Albooftileh, M. (2021). Preparation of biodegradable carboxymethyl cellulose-Arabic gum composite film and evaluation of its physical, mechanical and thermal properties. *Iranian Food Science and Technology Research Journal, 17*(2), 287-297.

Sahraee, S., Milani, J. M., Ghanbarzadeh, B., & Hamishehkar, H. (2017). Physicochemical and antifungal properties of bio-nanocomposite film based on gelatin-chitin nanoparticles. *International Journal of Biological Macromolecules, 97*, 373-381.

Shankar, S., Reddy, J. P., Rhim, J.-W., & Kim, H.-Y. (2015). Preparation, characterization, and antimicrobial activity of chitin nanofibrils reinforced carrageenan nanocomposite films. *Carbohydrate Polymers, 117*, 468-475.

***Images of different films and food model***


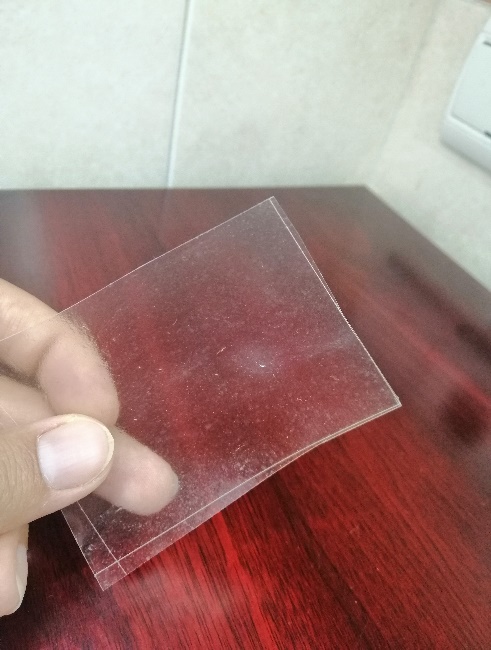

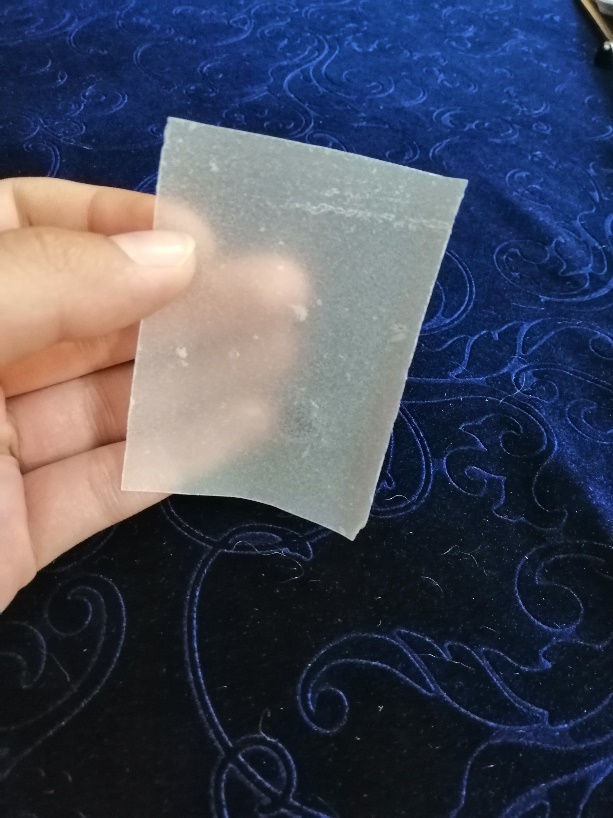
 hbh
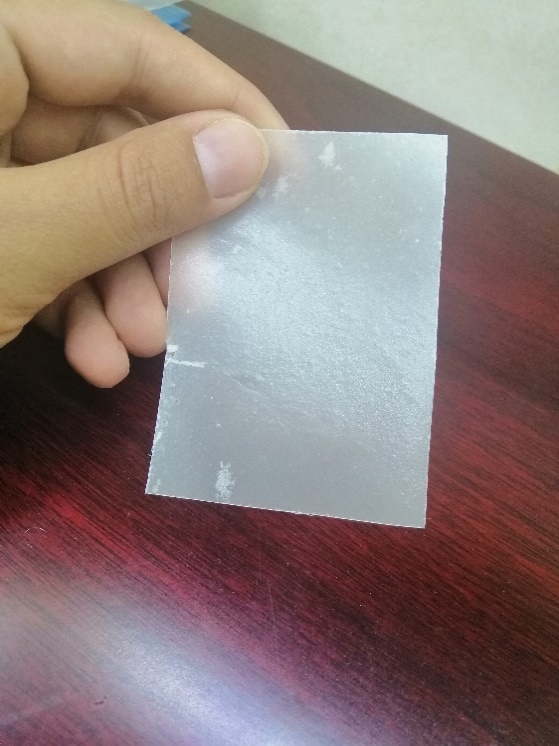


1. (B) (C)

A: TG-CNF film

B: TG-CNF-CEO film

C: TG-CNF-CNE film


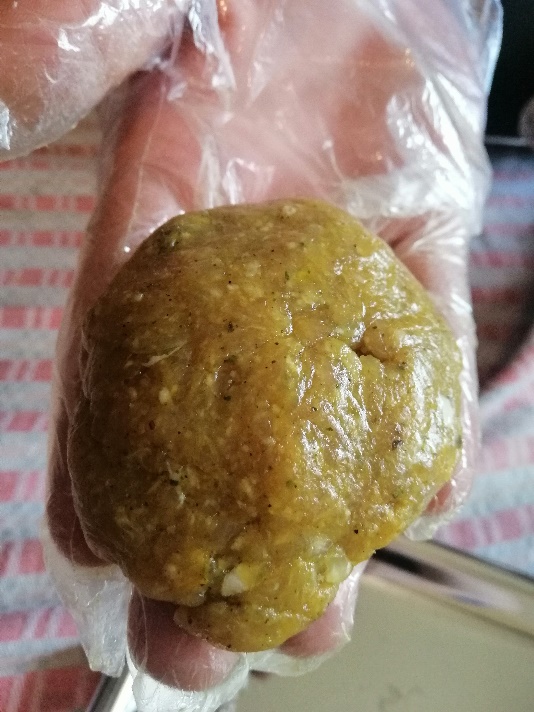

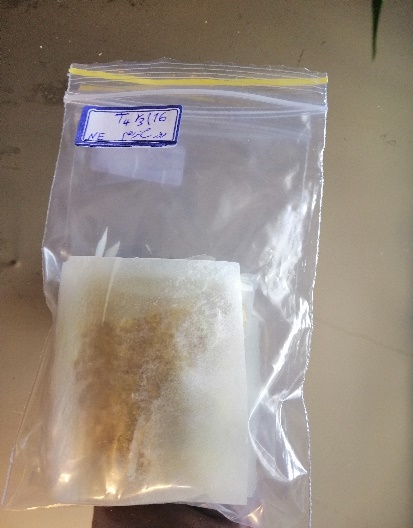


jjjk

(D) (E)

D: Turkey breast burger sample

E: Turkey breast burger sample wrapped in film
